# Exercise and GLUT4 in human subcutaneous adipose tissue

**DOI:** 10.14814/phy2.13918

**Published:** 2018-11-18

**Authors:** Marcelo Flores‐Opazo, Eva Boland, Andrew Garnham, Robyn M. Murphy, Sean L. McGee, Mark Hargreaves

**Affiliations:** ^1^ Department of Physiology The University of Melbourne Melbourne Australia; ^2^ Laboratory of Exercise and Physical Activity Sciences Department of Physiotherapy University Finis Terrae Santiago Chile; ^3^ School of Exercise & Nutrition Sciences Deakin University Burwood Australia; ^4^ Department of Biochemistry & Genetics LaTrobe Institute for Molecular Science LaTrobe University Melbourne Australia; ^5^ Metabolic Research Unit School of Medicine Deakin University Waurn Ponds Australia

**Keywords:** Adipose tissue, exercise, GLUT4, skeletal muscle

## Abstract

To examine the effect of acute and chronic exercise on adipose tissue GLUT4 expression, a total of 20 healthy, male subjects performed one of two studies. Ten subjects performed cycle ergometer exercise for 60 min at 73 ± 2% VO_2_ peak and abdominal adipose tissue samples were obtained immediately before and after exercise and after 3 h of recovery. Another 10 subjects completed 10 days of exercise training, comprising a combination of six sessions of 60 min at 75% VO_2_ peak and four sessions of 6 × 5 min at 90% VO_2_ peak, separated by 3 min at 40% VO_2_ peak. Abdominal adipose tissue and vastus lateralis muscle samples were obtained before training and 24 h after the last training session. A single bout of exercise did not change adipose tissue GLUT4 mRNA; however, there was a small, but significant, reduction in adipose tissue GLUT4 protein expression 3 h after exercise. There were no changes in adipose tissue GLUT4 or COX‐IV expression following exercise training. In contrast, skeletal muscle GLUT4 and COX‐IV were increased by 47% and 44%, respectively following exercise training. The exercise training‐induced increase in GLUT4 expression was similar in both type I and type IIa single muscle fibers. Our results indicate that neither a single exercise bout, nor 10 days of exercise training, increased adipose tissue GLUT4, in contrast with the increases observed in skeletal muscle GLUT4 expression.

## Introduction

The glucose transporter GLUT4 has a pivotal role in facilitating glucose uptake into skeletal and cardiac muscle in response to contractions and in adipose tissue and muscle in response to insulin stimulation. It has been previously demonstrated that both a single bout of exercise (Greiwe et al. 2000; Kraniou et al. 2000, 2006; McGee and Hargreaves 2004) and regular exercise training (Gulve and Spina 1995; Houmard et al. 1995; Phillips et al. 1996; Kraniou et al. 2004) increase GLUT4 expression in human skeletal muscle. Over the years, most attention has focused on skeletal muscle given its major role in whole‐body insulin‐mediated glucose disposal; however, adipose tissue is another important site of exercise‐mediated adaptations (Rodnick et al. 1987; Stanford et al. 2015). Given the importance of adipose tissue GLUT4 expression in mediating whole‐body glucose metabolism and insulin action (Herman and Kahn 2006), an increase in adipose tissue GLUT4 expression could contribute to the improved insulin action observed following exercise (Rodnick et al. 1987). Exercise training has been shown to increase adipose tissue GLUT4 protein expression in rodents (Hirshman et al. 1993; Stallknecht et al. 1993; Ferrara et al. 1998) but, to our knowledge, the effects of exercise on adipose tissue GLUT4 expression in healthy humans have not been examined. Thus, the aim of the present study was to assess the effects of acute and chronic exercise on GLUT4 protein abundance and gene expression in human subcutaneous adipose tissue.

## Methods

A total of 20 healthy, male subjects participated in two separate studies after providing informed, written consent. The study protocols were approved by the University of Melbourne Human Research Ethics Committee. At least 1 week prior to undertaking the studies, each subject undertook an incremental cycle ergometer (Lode, Groningen, The Netherlands) test to volitional fatigue to determine their peak pulmonary oxygen uptake (VO_2_ peak). Ten subjects (25 ± 1 year, 82 ± 3 kg, VO_2_ peak = 44 ± 2 mL·kg^−1^·min^−1^) completed an acute exercise study, while a second group of 10 subjects (27 ± 2 year, 75 ± 5 kg, VO_2_ peak = 42 ±  2 mL·kg^−1^·min^−1^) completed 10 days of exercise training.

### Experimental protocols

Subjects were instructed to consume their usual diet in the 24 h prior to the first experimental trial and reported to the laboratory in the morning after an overnight fast, having abstained from exercise, alcohol, and caffeine for 24 h. For the acute exercise study, subjects performed a single bout of cycle ergometer exercise at 73 ± 2% VO_2_ peak for 60 min, with water consumed ad libitum. Immediately before (PRE) and after (POST) the exercise bout, and after 3 h of passive recovery (3 h POST), subcutaneous, adipose tissue samples were obtained from the abdominal region (~5–10 cm lateral to the umbilicus) by percutaneous needle biopsy, with suction, under local anesthesia. Samples were washed in phosphate buffered saline, blotted on filter paper, and frozen in liquid N_2_ for later analysis. For the short‐term training study, pretraining (PRE) subcutaneous adipose tissue samples were obtained from the abdominal region and muscle samples were obtained from the vastus lateralis by percutaneous needle biopsy. Subjects then completed two blocks of five consecutive days of training comprising 60 min of cycling exercise at 75% VO_2_ peak on days one, three, and five, and 6 × 5 min cycling exercise bouts at 90% VO_2_ peak, with three min cycling at 40% VO_2_ peak between each bout, on days two and four. There was 1 day rest between the two training blocks. Posttraining adipose tissue and skeletal muscle samples (POST) were obtained 24 h after the last training session. The last training session included the posttraining VO_2_ peak test.

### Adipose tissue histology

In the training study, a fraction of the pre‐ and posttraining adipose samples from nine subjects was used for histology. Fresh samples were washed in PBS and placed in 10% formalin for 24 h. Samples were then dried and preserved in 70% ethanol until paraffin processing. Five 5 *μ*m thick tissue sections per sample were sliced with a microtome from paraffin blocks every 50 *μ*m and stained with hematoxylin‐eosin. Adipocyte size was determined at 10× magnification of stained sections using an automated system (Cell Profiler, Broad Institute, MA). Approximately, 300 cells per section, from three or four sections, were included in analyses.

### Adipose tissue GLUT4 gene expression

Total RNA was extracted from adipose tissue using an Aurum total RNA kit (Bio‐Rad, Hercules, CA). Samples were homogenized on ice in 1 mL PureZOL for ~30 sec. Samples were then incubated for 5 min at room temperature and spun at 4°C for 10 min at 10,000*g*. The supernatant was treated with 100% chloroform, incubated for 5 min at room temperature, and spun again at 4°C for 15 min at 10,000*g*. The aqueous phase was aspirated, treated with an equal volume of 70% ethanol and transferred to a mini‐RNA binding column. The samples were then treated with low stringency wash, spun for 3 sec at 10,000*g* at room temperature and then treated with DNase 1 for 15 min to remove genomic DNA. Samples were spun (30 sec, 10,000*g*), treated with high stringency wash, respun (30 sec, 10,000*g*), treated with low stringency wash and spun for 3 min at 10,000*g*. Elution of RNA from the binding column involved incubating samples in heated (70°C) elution solution for 1 min at room temperature, followed by a spin at 10,000*g* for 2 min. The final supernatant represented pure RNA and was stored at −80°C for later analysis. RNA was quantified spectrophotometrically at 260 nm and was free from genomic DNA (Experion, Bio‐Rad, Hercules, CA). Oligo dT single stranded cDNA was synthesized using an iScript cDNA synthesis kit (Bio‐Rad, Hercules, CA) in a thermocyler with a single cycle (5 min 25°C, 30 min 42°C, 5 min 85°C). Real‐time (RT) PCR was performed using SYBR Green reaction mix (Bio‐Rad, Hercules, CA) and primer sets complementary to human GLUT4 (FOR: 5′‐CTTCATCATTGGCATGGGTTT‐3′; REV: 5′‐AGGACCGCAAATAGAAGGAAGA‐3′) AND 18sRNA (FOR: 5′‐ACTGAGGATGAGGTGGAACG‐3′; REV: 5′‐TCCAGACCA TTGGCTAGGAC‐3′). RT PCR was undertaken in 25 *μ*L PCR plates in a iQ5 iCycler detection system with Bio‐Rad iQ5 Optical System Software (Bio‐Rad, Hercules, CA). The optimum annealing temperature for each primer set was assessed prior to each run and a nontemplate control for each primer was included. Each sample was subjected to melt curve analysis to detect primer‐dimers and nonspecific amplification. All samples displayed a single amplification product as indicated by a single peak. Expression of GLUT4 was assessed at all time points using the Δ−Δ CT method, relative to 18sRNA expression which did not change at any time point.

### Single muscle fiber isolation

A piece of frozen skeletal muscle tissue (~30 mg) was freeze‐dried (Freezone 4.5L benchtop freeze‐dry system, Labconco Corp. Kansas) for 48 h. Samples were stored in dessicant at −80°C until being brought to room temperature and single segments of muscle fibers were isolated under light microscope using forceps as previously described (Murphy 2011). Segments 1–3 mm in length were collected into 10 *μ*L loading buffer (1 × Lammelli′s buffer containing 10% *β*‐mercaptoethanol), briefly vortexed, incubated at room temperature for 1 h and stored at −80°C. A total of 30–60 single fiber segments were collected from each sample. The myosin heavy‐chain (MHC) isoform present in each muscle fiber segment was determined by dot blotting as previously described (MacInnis et al. 2017). Briefly, 1 *μ*L of each solubilized fiber sample was applied to a wet PVDF membrane following activation in 96% ethanol and equilibration in transfer buffer. The membrane was dried at room temperature, reactivated in 96% ethanol, blocked in 5% skim milk in TBST, and probed with specific MHC antibodies in the following order to qualitatively characterize the fibers as type IIa (MHCIIa; mouse monoclonal IgG, clone A4.74, Developmental Studies Hybridoma Bank [DSHB]; dilution 1:200 in 1% BSA/PBST) and type I (MHCI; mouse monoclonal IgM, clone A4.840, DSHBl dilution 1:200 in 1%BSA/PBST). Membranes were stripped between the two probes using a commercialized buffer (Pierce, Rockford, IL). For each sample, a minimum of nine fiber segments of a given MHC type were pooled prior to further immunoblotting.

### Adipose tissue and muscle protein extraction

In the acute exercise study, GLUT4 protein was assessed on total crude membrane fractions isolated from ~90 mg adipose tissue. Samples were homogenized (Polytron, Switzerland) in 1 mL of crude membrane lysis buffer (10 mmol/L Tris‐HCl pH 7.5, 1 mmol/L EDTA, 250 mmol/L sucrose, 2 *μ*L protease inhibitor complex) on ice for ~30–45 sec. The homogenate was spun (5 min, 10,000*g* at 4°C) to separate the fatty layer from the supernatant and the fatty layer was discarded. The supernatant was spun (60 min, 150,000*g* at 4°C) and the pellet resuspended in buffer (10 mmol/L Tris‐HCl pH 7.5, 1 mmol/L EDTA, 250 mmol/L sucrose, 10% SDS) and shaken under continuous agitation for 30 min. The solution was then spun (5 min, 10,000*g*, 4°C) and the final supernatant aspirated and stored at −80°C for later analysis. For determination of AMPK proteins, cell lysate protein was isolated from 50 to 70 mg of adipose tissue. Samples were homogenized in 10 volumes of whole cell lysate buffer (50 mmol/L Tris‐HCl pH 7.5, 1 mmol/L EDTA, 1 mmol/L EGTA, 10% glycerol, 1% Triton X‐100, 5 mmol/L sodium pyrophosphate, 1 mmol/L DTT, 2 *μ*L protease inhibitor complex) on ice for ~30–45 sec. The homogenate was spun (5 min, 10,000*g*, 4°C) and the supernatant aspirated and stored at −80°C for later analysis. In the exercise training study, ~200 mg of frozen adipose tissue were lysed in three volumes of lysis buffer (20 mmol/L HEPES pH 7.4, 2 mmol/L EGTA, 50 mmol/L *β*‐glycerophosphate, 1 mmol/L DTT, 1 mmol/L sodium orthovanadate, 10 *μ*L·mL^−1^ protease and phosphatase inhibitors) and homogenized using the Precellys 24 (Bertin Instruments, France) in two cycles at 4500*g* for 20 sec separated by 45 sec. Lysates were spun for 5 min at 5000*g* at 4°C. The upper fat layer was discarded and infranatants transferred to a new set of tubes and spun at 13,000*g* for 10 min at 4°C. Frozen muscle (20–30 mg) was homogenized (Polytron, 3 × 10 sec burst on ice) in 20:1 v/w of physiological buffer (90 mmol/L HEPES, 50 mmol/L EGTA, 8 mmol/L Na_2_ATP, 10 mmol/L Na_2_Creatine Phosphate, pH 7.1), supplemented with protease inhibitors (CompleteMini^®^; Roche Applied Science, Laval, Canada). The final concentration of the preparation was 50 *μ*g·*μ*L^−1^. Next, 100 *μ*L of whole muscle homogenate were transferred to prechilled microtubes containing 50 *μ*L 3 × SDS. Following incubation for 1 h at room temperature, samples were frozen at −80°C for 2 h, thawed to room temperature and a working stock prepared by diluting 10 *μ*L sample to a final concentration of 2.5 *μ*g·*μ*L ^−1^ with 1 × SDS. A pooled sample was prepared by mixing equal amounts of each sample and used for calibration curves on all gels. Total protein in adipose tissue and muscle samples was measured using the BCA method (Pierce, Rockford, IL).

### Immunoblotting

The samples were subjected to SDS‐PAGE and immunoblotting with specific antibodies to assess GLUT4 (acute study: anti‐rabbit polyclonal, 1:750, Biogenesis, UK; training study: anti‐rabbit, polyclonal 1:1000, Thermo Fisher Scientific) and COX‐IV (anti‐rabbit, polyclonal, 1:1000, Cell Signaling, Danvers, MA). In the acute exercise study, adipose tissue samples obtained immediately before and after exercise were probed for AMPK and phospho‐^Thr172^AMPK (1:500, Cell Signaling, Danvers, MA). After overnight incubation in primary antibodies, membranes were washed 3 × 10 min in TBST and exposed to secondary antibody for 1 h at room temperature. Membranes were washed again three times in TBST and the adipose tissue membranes incubated for 1 h in chemiluminescence reagents (GE Healthcare/Amersham, Uppsala, Sweden) or the whole muscle and single fiber muscle membranes for 1 min using West Femto chemiluminescent reagent (Therm Scientific, Australia). Antibody binding was visualized, imaged, and quantified using the ChemiDoc MP system and Image Lab software (Bio‐Rad, Hercules, CA). The density of the proteins of interest was normalized relative to *β*‐actin (adipose samples) as loading control or total protein, as quantified from Stain‐free gels (muscle samples).

### Statistics

Due to technical difficulties during analysis of samples from the acute exercise study, results were not obtained for all subjects and actual sample numbers are reported as appropriate. Data from the two experiments were compared using paired *t*‐test or analysis of variance, as appropriate, with significance at the *P *<* *0.05 level. Data are reported as means ± SEM.

## Results

**Figure 1:**
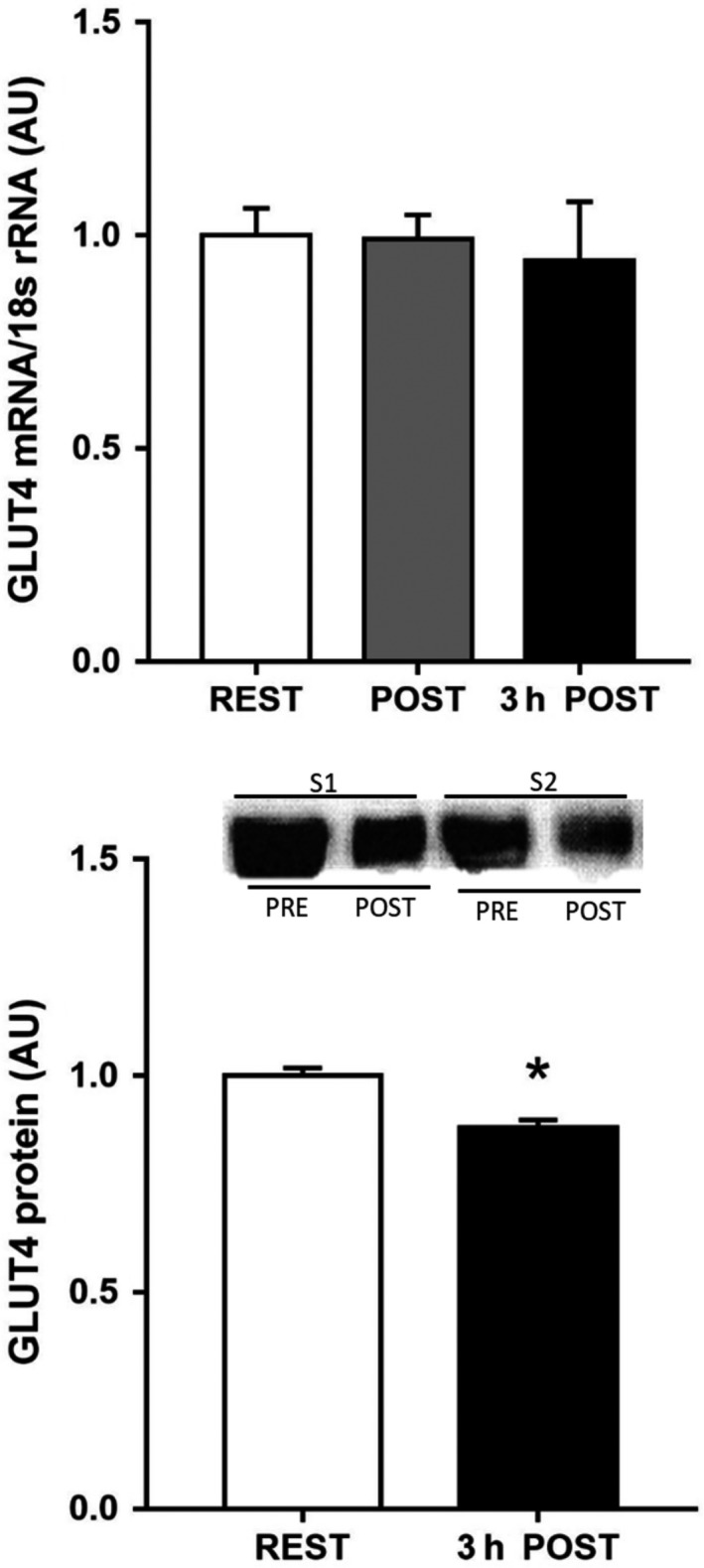
GLUT4 mRNA expression before (PRE), immediately post (POST) and 3 h post (3 h POST) 60 min exercise at 73 ± 2% VO_2_ peak, and GLUT4 protein expression (bottom panel) before and 3 h after exercise, in human, subcutaneous adipose tissue. Data are means ± SEM (*n* = 5 for mRNA and *n* = 7 for protein). * denotes different from PRE (*P* < 0.05–0.10).

**Figure 2:**
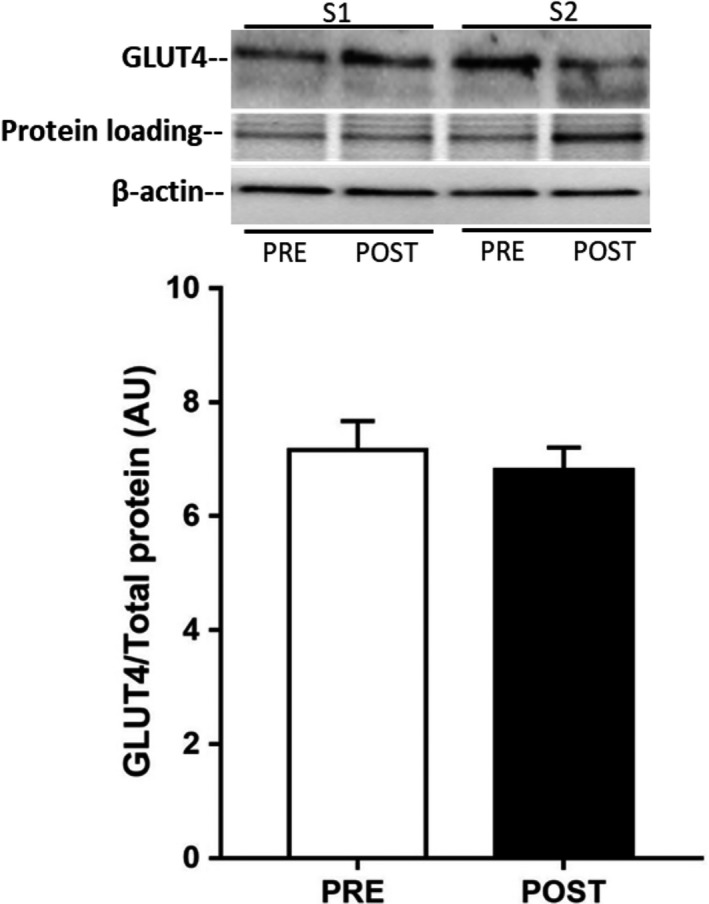
GLUT4 protein expression in human subcutaneous adipose tissue before (PRE) and after (POST) 10 days of exercise training. Data are means ± SEM (*n* = 10).

**Figure 3:**
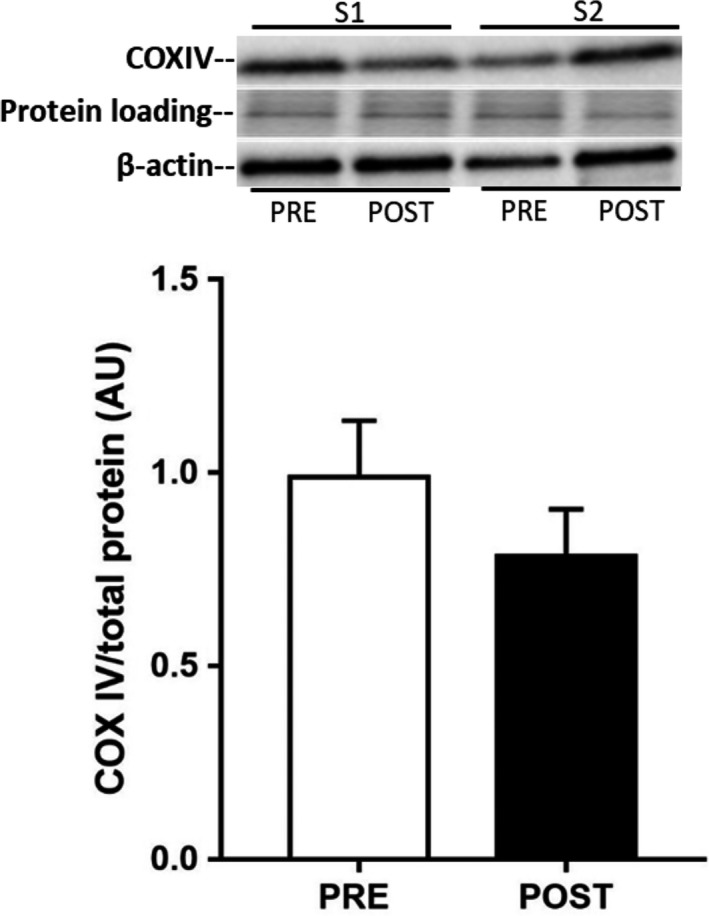
COX‐IV protein expression in human subcutaneous adipose tissue before (PRE) and after (POST) 10 days of exercise training. Data are means ± SEM (*n* = 10).

**Figure 4:**
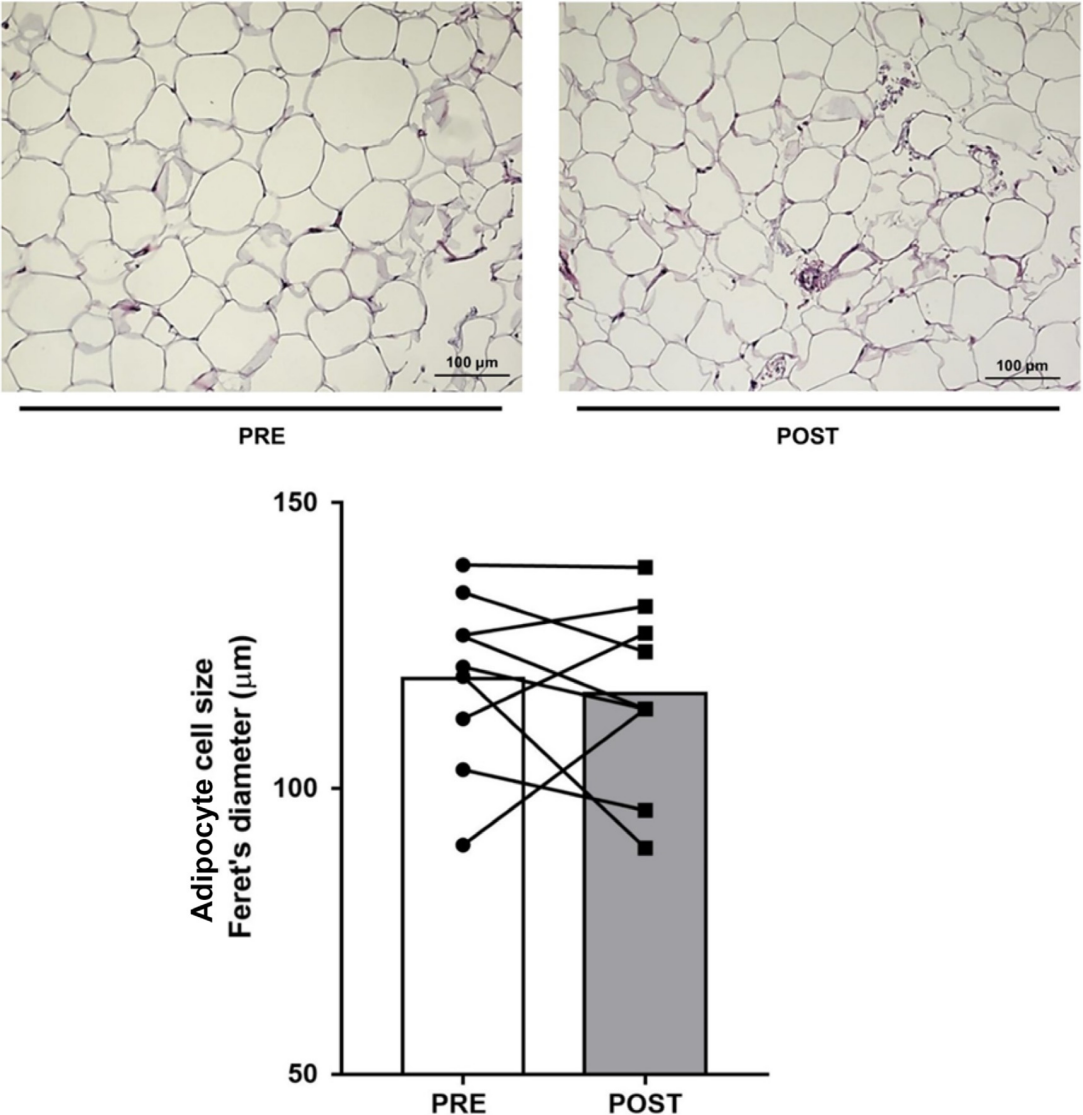
Representative micrographs of adipose tissue histology (top panels) and mean cell diameter (bottom panel) before (PRE) and after (POST) 10 days of exercise training. Individual responses and means shown.

**Figure 5:**
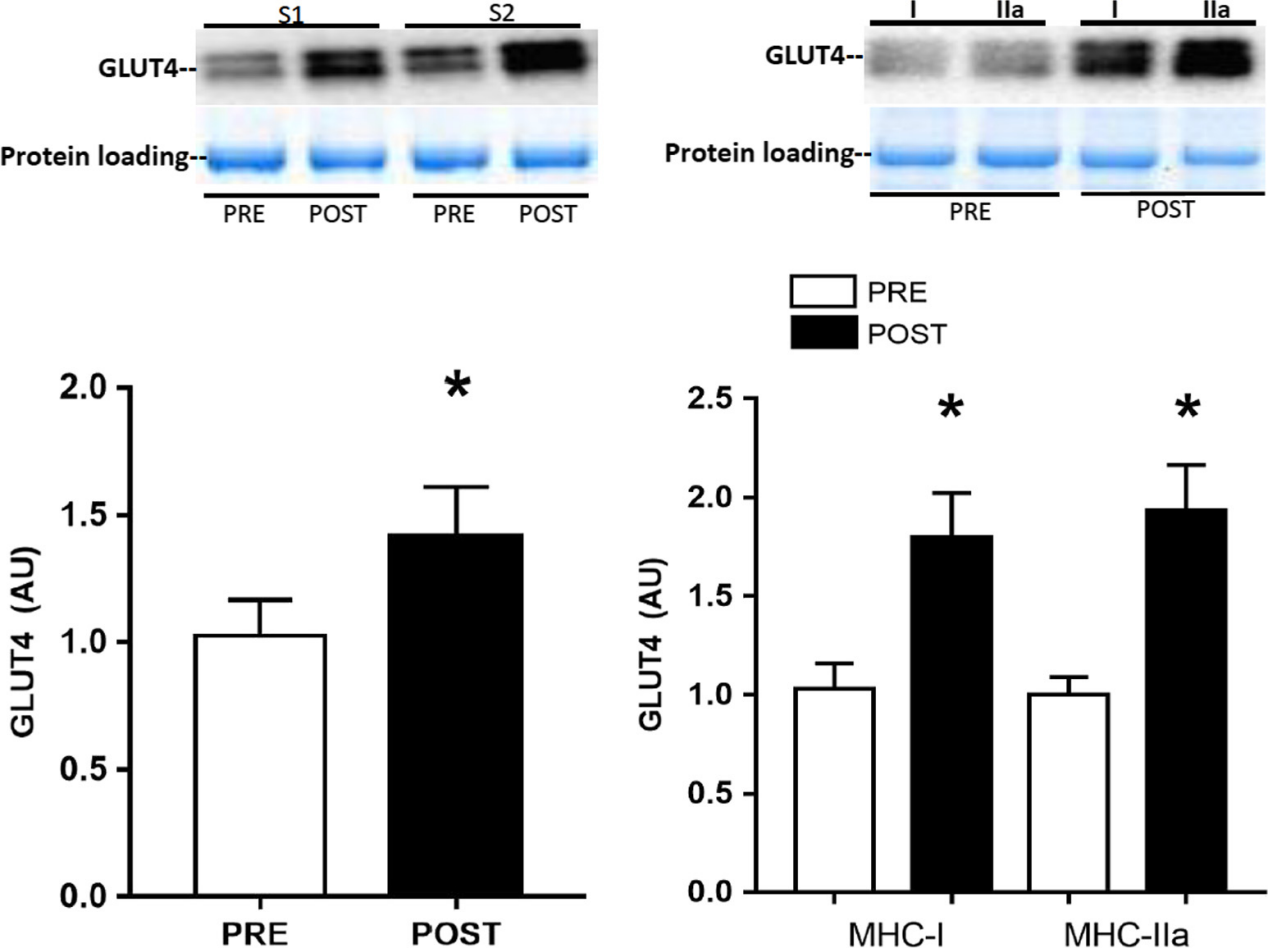
GLUT4 protein expression in whole skeletal muscle (left panel) and pooled single type I and IIa muscle fibers (right panel) before (PRE) and after (POST) 10 days of exercise training. Data are means ± SEM (*n* = 10). * denotes different from PRE (*P* < 0.05).

A single bout of exercise did not change adipose tissue GLUT4 mRNA (Fig. 1); however, there was a small, but significant, reduction in adipose tissue GLUT4 protein expression 3 h after exercise [0.88 ± 0.02 vs. 1.00 ± 0.02 arb units, *P *=* *0.043 (one‐tail), *P *=* *0.086 (two‐tail), Fig. 1]. Neither total AMPK (PRE: 1.00 ± 0.15 vs. POST: 1.04 ± 0.06 arb units, *n* = 6) nor phospho‐^Thr172^AMPK (PRE: 1.00 ± 0.13 vs. POST: 0.89 ± 0.09 arb units, *n* = 6) expression were affected by exercise, nor was the phospho‐^Thr172^AMPK:AMPK ratio (PRE: 1.0 ± 0.1 vs. POST: 0.9 ± 0.2 arb units, *n* = 6). Exercise training increased VO_2_ peak by 5% (POST: 44 ± 3 mL·kg^−1^·min^−1^ vs. PRE: 42 ± 2 mL·kg^−1^·min^−1^, *P* < 0.05), with no change in body mass, and peak power by 6% (POST: 275 ± 14 vs. PRE: 260 ± 13 W, *P* < 0.05). There were no changes in adipose tissue GLUT4 (Fig. 2) and COX‐IV expression (Fig. 3), or in adipocyte cell size (Fig. 4), following exercise training. In contrast, skeletal muscle GLUT4 (Fig. 5) and COX‐IV (PRE:1.6 ± 0.3 vs. POST: 2.3 ± 0.3 arb units, *P* < 0.05) were increased by 47% and 44%, respectively, following exercise training. The exercise training‐induced increase in GLUT4 expression was similar in both type I and type IIa pooled, single muscle fibers (Fig. 5).

## Discussion

The results of the present study suggest that adipose tissue GLUT4 may be less responsive to exercise stimuli than skeletal muscle GLUT4, at least in terms of increased protein expression. A single exercise bout did not change adipose tissue GLUT4 mRNA expression; however, 3 h after exercise there was a small, but significant, reduction in adipose tissue GLUT4 protein content. We have no data on rates of GLUT4 synthesis and degradation in adipose tissue following exercise, but our observation suggests there must have been alteration in one or both resulting in the slightly lower GLUT4 protein content 3 h postexercise. It is possible that such a response serves to reduce adipose tissue glucose uptake and facilitate preferential glucose uptake into skeletal muscle for glycogen resynthesis in the postexercise period. This is speculative and further work is required to test such a hypothesis. In any event, we believe this response is probably transient as we did not observe a reduction is adipose tissue GLUT4 protein levels after 10 days of exercise training, when measured 24 h after the last training session (Fig. 2). We (Kraniou et al. 2000, 2006; McGee and Hargreaves 2004) and others (Greiwe et al. 2000) have previously observed that a single exercise bout increases skeletal muscle GLUT4 mRNA and protein expression. GLUT4 expression is regulated via the HDAC‐MEF2 axis (McGee and Hargreaves 2004) and a key upstream kinase mediating exercise effects on GLUT4 expression is AMPK (McGee et al. 2008). In the present study, there was no effect of exercise on the phospho‐^Thr172^AMPK:total AMPK ratio, a proxy for AMPK activation, in adipose tissue. Previous studies examining AMPK activity in human adipose tissue during exercise have produced equivocal results (Watt et al. 2006; Kristensen et al. 2007). However, it would appear that exercise of the intensity and duration utilized in the present study did not activate AMPK in human subcutaneous adipose tissue.

Our rationale for undertaking the exercise training study was to determine whether a greater exercise stimulus would increase adipose tissue GLUT4. Again, however, there was no effect of 10 days of exercise training on adipose tissue GLUT4 content, or oxidative capacity as measured by COX‐IV expression, despite the ~45% increase in both these proteins in skeletal muscle in the present study and the well described increases in skeletal muscle GLUT4 content with short‐term training (Gulve and Spina 1995; Houmard et al. 1995; Phillips et al. 1996; Kraniou et al. 2004). Our results are consistent with a previous study that observed no effect of 10 days of exercise training on the oxidative capacity of human subcutaneous adipose tissue (Camera et al. 2010). It is possible that a longer duration of exercise training is required to elicit changes in GLUT4 content and oxidative capacity in human adipose tissue. That said, a 6 weeks high‐intensity training protocol also did not increase adipose tissue oxidative capacity in humans (Larsen et al. 2015). Previous studies in rodents have demonstrated exercise training‐induced increases in GLUT4 and other metabolic biomarkers (Hirshman et al. 1993; Stallknecht et al. 1993; Ferrara et al. 1998; Stanford et al. 2015) associated with so‐called “browning.” It may be that human adipose tissue is relatively less responsive to exercise training compared to rodents, at least in terms of some of these metabolic adaptations (Norheim et al. 2013). Differences in the fat store (subcutaneous vs. visceral) studied and the fact that laboratory rodents are often housed at temperatures lower than thermoneutrality may be other potential explanations for the divergent adipose tissue GLUT4 response to exercise between humans and rodents.

Adipose tissue GLUT4 content appears to be important for whole‐body glucose metabolism and insulin action (Herman and Kahn 2006). Selective deletion of GLUT4 in adipose tissue results in muscle and hepatic insulin resistance (Abel et al. 2001), potentially via increased plasma levels of retinol binding protein 4 (RBP4, Yang et al. 2005) or other adipokines. Of note, adipose tissue from patients with obesity and type 2 diabetes has reduced GLUT4 expression (Garvey et al. 1991; Sinha et al. 1991). Overexpression of GLUT4 in adipose tissue results in improved glucose tolerance (Shepherd et al. 1993) and enhanced insulin action (Carvalho et al. 2005), despite increased fat cell mass. We have previously observed that 4 weeks of exercise training in patients with type 2 diabetes increased adipose tissue GLUT4 expression to levels similar to those in age‐, BMI‐matched healthy subjects (Hussey et al. 2011). Furthermore, although exercise effects on adipose tissue GLUT4 content were not measured directly, an effect was inferred from an exercise training‐induced reduction in serum RBP 4 levels and the inverse relationship between serum RBP4 and adipocyte GLUT4 (Graham et al. 2006). Thus, it is possible that exercise training is more effective in increasing adipose tissue GLUT4 content when pretraining GLUT4 expression levels are reduced, as is the case in type 2 diabetes.

In the present study, we observed no difference in GLUT4 protein content between type I and IIa single muscle fibers, either before or after training. This contrasts slightly with the ~20% higher GLUT4 content in type I fibers reported previously (Daugaard et al. 2000). The GLUT4 expression across human skeletal muscle fiber types is much more similar, compared to the larger differences reported in rodent muscle fibers, and it may not always be possible to detect significant differences in human samples. It has also been reported that 2 weeks of exercise training at 40% Wmax increased GLUT4 protein content in the type I, but not the IIa or IIx, muscle fibers (Daugaard et al. 2000). In the present study, we observed increased GLUT4 protein content in both type I and IIa fibers following 10 days of exercise training, most likely a consequence of the fiber recruitment patterns associated with the higher exercise intensities used during the training protocol.

In summary, neither a single exercise bout, nor 10 days of exercise training, increased adipose tissue GLUT4 mRNA or protein content, in contrast with the consistent increases in skeletal muscle GLUT4 expression observed with such interventions.

## Conflict of Interest

None declared.
